# Intersectional Barriers Among PLHIV in Rural Illinois: Insights from a Pilot QCA Study

**DOI:** 10.3390/ijerph22071011

**Published:** 2025-06-26

**Authors:** John Matta

**Affiliations:** School of Engineering, Southern Illinois University Edwardsville, Edwardsville, IL 62025, USA; jmatta@siue.edu

**Keywords:** health equity, SDoH, HIV/AIDS, social stigma

## Abstract

People living with HIV (PLHIV) in under-resourced rural regions face intersecting social and structural barriers that intensify their vulnerability. This pilot study explored how overlapping marginalized identities and socioeconomic hardship shape experiences of discrimination among PLHIV in Southern Illinois. Twenty-two participants completed a community-informed survey that captured racial, sexual, and gender identities alongside indicators of stigma across healthcare, housing, employment, and community settings. The findings reveal that most participants experienced at least one form of discrimination, with the highest burden concentrated among those identifying as Black, Nonbinary, Gay/Lesbian, or low-income. Discrimination was particularly prevalent in healthcare and housing environments. Importantly, poverty and housing instability were not just common but appeared to amplify the experience of stigma, compounding the effects of identity-based marginalization. These results highlight the urgent need for integrated, affirming, and structurally responsive interventions tailored to the realities of multiply marginalized PLHIV in rural areas. Future research and services must consider the interplay of identity and economic precarity in order to promote equitable care and support.

## 1. Introduction

Despite advances in HIV treatment, people living with HIV (PLHIV) continue to face stark disparities in care access and outcomes, particularly in rural U.S. regions. These disparities are driven not only by clinical factors, but also by social determinants of health (SDoH) [1], including housing instability, poverty, and stigma from healthcare systems and communities. The rural communities of Southern Illinois exemplify this context. It is a region marked by economic disinvestment, healthcare fragmentation, and inconsistent access to LGBTQ^+^-affirming care.

Intersectionality theory provides a framework to understand how overlapping identities such as race, gender identity, and sexual orientation produce unique experiences of stigma and exclusion. In public health, this framework has been used to analyze compounded risks faced by marginalized groups [2,3,4], but it remains underutilized in rural settings. This study therefore asks the following question: How do intersecting identities shape the experiences of discrimination for PLHIV in Southern Illinois? We address this question using an intersectionality-informed analytical approach.

Rural and semi-urban regions are often marked by healthcare provider shortages, economic precarity, and persistent stigma, all of which affect access to HIV care and prevention services. For example, a statewide assessment by McLuckie et al. [5] found that only 13.2% of rural Illinois health departments offered PrEP, despite 70% offering HIV testing. The current author’s *Burden of HIV* survey [6] similarly found no respondents using PrEP, underscoring persistent gaps in prevention access even among MSM communities [7]. Research indicates that transgender individuals [8] and sex workers [9] often experience even greater obstacles to care.

This study addresses a critical gap by applying an intersectionality-informed and pattern-based analytical approach to the experiences of PLHIV in Southern Illinois. Using data from the *Burden of HIV* survey, which employed respondent-driven sampling (RDS) [10] to reach HIV-positive individuals and their partners, we examine how combinations of racial, sexual, and gender identities correspond with multiple forms of discrimination across several domains. While many varying strategies have been used in HIV research, including network science tools such as betweenness centrality [11] and homophily [12], machine learning techniques such as clustering [13], and statistical analysis [14], here, we use Qualitative Comparative Analysis (QCA) [15] to identify consistent identity-based configurations of stigma and complement these findings with visual and composite burden analyses. QCA offers a distinct advantage in small-sample research by identifying complex, non-linear, and combinatorial relationships that may not be detectable through variable-centered methods. QCA is particularly well suited for intersectional analyses because it accommodates multiple causal pathways and reveals how specific configurations of conditions (e.g., Black + Bisexual) are consistently associated with outcomes such as stigma. This allows researchers to go beyond bivariate associations and explore how intersecting identities operate together to shape experiences of exclusion.

Rather than seeking generalizability, this study prioritizes descriptive insight and intersectional pattern recognition. By focusing on a historically under-researched region, we contribute to the evidence base for equity-oriented, structurally responsive HIV interventions in rural America. These findings also support broader theoretical trends in public health that emphasize multilevel models of vulnerability, such as the syndemic framework [16] and structural competency theory [17], by showing how intersecting social identities, compounded structural exclusions, and contextual resource deprivation jointly shape health outcomes for PLHIV. Our study reaffirms that HIV-related disparities cannot be meaningfully addressed without situating individual experiences within the structural and cultural forces that shape them.

## 2. Related Work

### 2.1. Theoretical and Intersectionality Frameworks

The theoretical foundation for this study is rooted in intersectionality, probably best known through the work of Crenshaw [18], which describes how overlapping systems of oppression create distinct experiences of marginalization. In framing the theoretical foundation of intersectionality, it is important to acknowledge its intellectual roots that precede Crenshaw’s legal scholarship. Key contributions from Black feminist theorists, particularly Patricia Hill Collins and Sirma Bilge, articulate how intersecting identities, such as race, class, and gender, are deeply intertwined and shape individual experiences of oppression. In their work *Intersectionality* [19], Collins and Bilge emphasize the importance of viewing social categories as dynamic and relational rather than additive, which enhances our understanding of how discrimination manifests among diverse populations [20]. Bilge further critiques the trend of appropriating intersectional discourse without engaging its radical roots, insisting that it must retain its political significance [21].

Bowleg [22] expands on this framework in public health, arguing for its necessity in addressing health inequities that extend beyond individual-level risks. This perspective has been particularly influential in HIV research involving Black Gay and Bisexual men, transgender individuals, and Black women, as evidenced by the work of Stangl et al. [23], who highlighted how intersectional stigma affects treatment engagement among these groups. Additionally, McConnell et al. [24] further illustrate the compounded nature of stigma faced by sexual minority men of color, emphasizing the need for interventions that consider the intersections of identity in combating health disparities.

### 2.2. Stigma and Multilevel Barriers in HIV Care

A substantial body of research demonstrates that PLHIV experience compounded stigma shaped by race, gender identity, sexual orientation, and socioeconomic status. Turan et al. [25] developed a multilevel framework illustrating how HIV stigma undermines treatment engagement and mental health, emphasizing that stigma arises not only from individual attitudes but also from structural inequalities. Logie et al. [26] indicate that intersecting stigmas significantly worsen health outcomes among racial, sexual, and gender minorities, highlighting how these overlapping identities contribute to poorer health trajectories [27]. Furthermore, Rice et al. specifically examined the experiences of diverse women living with HIV and found that societal drivers of HIV stigma create significant barriers to care and treatment, reinforcing the need for targeted interventions that address these compounded vulnerabilities [28].

### 2.3. Geographic and Population Context

Recent empirical work extends intersectional frameworks into rural and under-resourced settings. A study by Quinn et al. describes how PLHIV in rural areas experience systemic challenges, including limited transportation, healthcare stigma, and economic hardship, which can exacerbate feelings of isolation and depression among older adults [29]. Endres et al. explored stigma navigation strategies among people who inject drugs (PWID) in Appalachia, revealing a reliance on peer support and resilience-building as vital coping mechanisms in the face of intersecting stigmas [30]. While Bergo et al. examine vulnerabilities in regard to injection drug use, it is important to note that their focus is on broader health-related predictors rather than the layered stigma faced by specific demographics [31]. Scott et al. highlight that Black women in the South face layered stigma rooted in race, gender, and HIV status, which significantly contributes to healthcare avoidance and housing insecurity [32].

### 2.4. Intervention Models and Structural Approaches

To address these inequities, several interventions have been developed that integrate identity-informed and structural frameworks. The “Black Women First” initiative, as described by Walter et al. [33], implemented bundled care models that include trauma-informed services, housing assistance, and legal advocacy, resulting in improved care retention among Black women. These models illustrate how intersectionality-informed programming can be implemented in real-world clinical and community contexts.

### 2.5. QCA and Methodological Approaches in Social Research

Qualitative Comparative Analysis (QCA) has emerged as a powerful method for analyzing complex causal relationships in small-N settings. It is particularly useful in public health and social science research, where intersecting identities and social conditions influence outcomes [34]. QCA allows for the identification of distinct combinations of conditions that can lead to the same outcome, recognizing that various pathways may produce similar results [35]. In HIV research, QCA has been employed to investigate treatment adherence, care engagement, and structural vulnerability, shedding light on how different contextual factors intersect to affect health outcomes [36]. Best practices for applying QCA in applied research include careful calibration, model parsimony, and theoretical alignment, all of which are crucial for ensuring rigor in analysis [37]. By applying QCA to a rural Midwestern population of people living with HIV (PLHIV), our study extends this method to identify identity-based configurations linked to discrimination encountered in healthcare, housing, and social contexts.

### 2.6. Positioning This Study

Together, these studies reinforce the necessity of intersectional and structurally responsive approaches to HIV care, especially in under-resourced settings. Our study builds on this literature by combining intersectionality theory, visual synthesis, and QCA to explore how identity configurations influence multi-domain discrimination. In doing so, we contribute new insights to the ongoing effort to contextualize stigma and inform equity-focused interventions in rural HIV care.

## 3. Methods

### 3.1. The Burden of HIV Survey

Data were collected from the *Burden of HIV* survey, described in Matta et al. [6]. This cross-sectional pilot study, conducted from late 2021 to 2023, tried to identify patterns of social and structural barriers faced by PLHIV in Southern Illinois, a region with limited access to LGBTQ^+^-affirming care. Pilot studies such as this are essential for testing instruments and exploring conceptual frameworks, particularly in under-researched populations where exploratory analysis is appropriate [38]. The study design was grounded in intersectionality theory and the social determinants of health framework.

Twenty-two participants were recruited via community-based outreach and local HIV service providers. Eligible individuals were aged 18 or older, lived in the Southern Illinois region, and were at least one of the following: HIV-positive, men who have sex with men (MSM), an injecting drug user (IDU), or the partner of an MSM, IDU, or HIV-positive person. The Applied Research Consultants (ARC) group at Southern Illinois University Carbondale programmed and administered the survey. The instrument began with an informed consent form, which included contact information for the survey administrator and listed local HIV-related care resources. To protect participant anonymity, ARC implemented established data security protocols. Identifying information was removed following survey completion. Participants received compensation for their participation and were invited to refer others for additional incentives, making this a respondent-driven sampling (RDS) survey [39].

While the RDS framework permits peer recruitment, few participants referred others. To ensure representation of diverse identities for intersectional analysis, purposive recruitment was used to include variation in race, gender identity, sexual orientation, and income [40]. Although the sample is small, it was intentionally designed to capture a broad range of experiences relevant to intersectionality. In theory, RDS yields an unbiased sample if referral chains are sufficiently long. However, because recruitment chains in this study were short, we do not assume sample representativeness and refrain from applying inferential statistical methods.

This survey measures perceived discrimination, economic hardship, social support, and COVID-19 pandemic impacts. It draws its questions from validated measures used in two prior HIV stigma and structural vulnerability research surveys [41,42]. The key domains included the following:

- Demographics: Race, gender identity, sexual orientation, housing, and income.
- Discrimination: Thirty-three types of discrimination are included, based on all combinations of educational, transportation, housing, healthcare, job, social and community discrimination, combined with race, sexual orientation, HIV status, and drug use. Additionally, discrimination based on credit and criminal record in housing; trans status in healthcare; and trans and educational status in employment were tallied.
- Social support: HIV disclosure, social isolation, and perceived family support.
- COVID-19 effects: Job loss, housing instability, and behavior changes.

All variables were transformed into binary indicators using one-hot encoding, a common strategy for enabling categorical pattern recognition and exploratory modeling in small-N public health and mixed-methods research [43].

Income was divided into below USD 15,000 and above USD 15,000. An income of USD 15,000 was used to determine “low income” status, as the United States Health and Human Services poverty level for the year of the survey (2023) is USD 14,580. Binary variables for low income and housing instability (homelessness, temporary housing, or COVID-related decline) were cross-tabulated with aggregated discrimination outcomes to assess compounding effects of economic hardship [44,45]. A binary summary variable was computed to capture whether any form of discrimination or social isolation was reported, reflecting the multifaceted and layered nature of stigma and exclusion experienced by PLHIV, consistent with prior composite stigma research [46].

### 3.2. Analytical Strategy and Best Practices Application

Due to the exploratory nature and small sample size (n=22), traditional inferential statistics were not used. Instead, we applied a pattern-based analytic framework rooted in QCA- and case-based logic [47]. This approach allows for the examination of configurational conditions (e.g., identity intersections) associated with outcomes such as discrimination and social exclusion. Participants were grouped by binary indicators (Black, Gay/Lesbian, Bisexual, Nonbinary), and QCA-style truth tables were generated to explore the relationship between identity intersections and reported discrimination. This methodology has been recommended for intersectionality-informed health equity research [48,49].

QCA is well suited to small-N studies where the goal is to identify patterns across intersecting conditions. Best practices in small-N QCA emphasize theoretical grounding in condition selection, transparent calibration, manageable model complexity, and rigorous truth table interpretation [50]. Following these standards, our study began with theory-driven selection of identity-based conditions. We limited the number of causal conditions relative to the number of cases to avoid overfitting and retained only the most theoretically salient variables for QCA modeling. Calibration was based on binary indicators derived from survey responses. Truth tables were constructed for multiple discrimination outcomes across distinct domains (e.g., healthcare, housing, employment), and contradictions were examined in context. To mitigate the risk of overfitting and evaluate the robustness of results, we assessed both consistency and coverage for the identified configurations. For example, in the analysis of employment discrimination, the configuration identifying as both Black and Gay/Lesbian demonstrated perfect consistency (1.0) and an estimated coverage of 0.77, suggesting that it may be a sufficient condition in this context. Similarly, in analyses of healthcare discrimination due to race, the same identity configuration showed a consistency of 0.71. In contrast, configurations involving socioeconomic variables (e.g., low income and housing instability) did not meet the conventional consistency threshold of 0.67 and were not considered sufficient. We further contextualized QCA results with reference to individual case characteristics, supported by heatmaps and composite burden scores. Taken together, these practices demonstrate a rigorous and transparent application of QCA that highlights intersectional patterns of stigma among PLHIV in a rural U.S. setting.

## 4. Results

In the results that follow, percentages are accompanied by raw counts in parentheses to aid interpretation, particularly given the small sample sizes and the resulting variability in estimate stability.

### 4.1. Participant Characteristics

The final sample included 22 respondents. Participants represented a range of identities: 68% (15) identified as Black, 50% (11) as Gay or Lesbian, 18% (4) as Bisexual, and 9% (2) as Nonbinary. Income levels were low overall, with 59% (13) reporting annual incomes below USD 15,000. In terms of housing, 68% (15) experienced some form of instability, including homelessness, temporary housing, or COVID-related disruptions.

### 4.2. Prevalence of Discrimination

Discrimination occurred at high levels. Seventeen participants (77%) reported experiencing at least 1 of the 33 forms of discrimination. This result is not unexpected, as the survey’s inclusion criteria specifically targeted individuals from groups commonly subject to discrimination, including PLHIV, MSM, and individuals who inject drugs. The burden of discrimination was reported across multiple domains including housing, healthcare, employment, educational, and community settings. Composite analysis revealed frequent co-occurrence of multiple discrimination types, underscoring the importance of analyzing overlapping and intersecting experiences.

### 4.3. Intersectional Identity Patterns: Core Discrimination Domains

A QCA-style truth table (as shown in Table 1) presents all observed combinations of binary conditions (e.g., identity attributes) and their associated outcomes (e.g., experiences of discrimination). Each row represents a unique configuration of conditions across participants—for example, individuals who are both Black and Bisexual (and not Gay/Lesbian or Nonbinary). Columns on the left list the presence (1) or absence (0) of each identity, while columns on the right show the outcome(s) observed for that configuration. This format allows for the identification of which identity combinations are consistently associated with specific outcomes and which are not, highlighting both patterns of marginalization and potential exceptions.

Several patterns emerge. Notably, individuals identifying as both Black and Gay/Lesbian exhibited the highest prevalence of reported discrimination, particularly community-based (86%) and race-based healthcare discrimination (71%). In contrast, configurations involving single minority identities, such as being Nonbinary alone or Bisexual alone, showed lower and more variable rates of discrimination. These findings support the interpretation that intersectional identities are associated with compounded forms of marginalization, particularly within healthcare and community settings, aligning with the theoretical expectations of intersectionality theory. However, the overall sparsity of some configurations underscores the small-sample, exploratory nature of the study.

### 4.4. Separate Pattern in Employment and Social Exclusion Domains

To extend the analysis, we examined employment and social discrimination as outcomes using a second QCA-style truth table, presented in Table 2. The two outcome measures were constructed by summing binary indicators of job-related discrimination (e.g., discrimination that kept the participant from obtaining or holding a job) and social discrimination (e.g., being treated unfairly by social services).

Overall, high levels of discrimination were observed across most identity groupings, but especially among those with multiple marginalized identities. Participants identifying as both Black and Gay/Lesbian experienced the highest rates of job discrimination (100%) and high levels of social discrimination (86%). Similarly, all participants who identified as Nonbinary, whether in combination with other identities or not, reported both job and social discrimination. In contrast, participants who identified solely as Black had more variability, with only 20% reporting job discrimination but 40% reporting social discrimination.

Groups with lower sample counts, such as those identifying as Gay/Lesbian and Nonbinary, still reported discrimination, underscoring the breadth of stigma even in small or less-represented subgroups. These findings reinforce the intersectional nature of discrimination burden, demonstrating that the accumulation of marginalized identities corresponds with elevated risks of structural and interpersonal stigma.

When comparing Table 2 with Table 1, a key distinction emerges. In Table 1, several identity configurations, particularly those involving Black and Gay/Lesbian participants, were clearly associated with multiple domains of discrimination. In contrast, Table 2 shows that reports of employment and social discrimination are high across almost all identity groups. This suggests that while intersectional identity combinations strongly predict disparities in some settings (e.g., healthcare), other domains, such as employment, may reflect more generalized or diffuse forms of exclusion that cut across identity categories. These findings support the need for both intersectional and domain-specific analyses in understanding the full scope of structural stigma experienced by PLHIV.

### 4.5. Socioeconomic Status and Amplification Effects

We assessed whether structural hardship (e.g., low income or housing instability) amplified discrimination. The QCA-style truth table (Table 3) indicates that structural hardship, defined as the combination of low income and housing instability, may be associated with increased exposure to certain types of discrimination, particularly healthcare discrimination due to race and community-level HIV stigma. These findings suggest that even within a broadly marginalized sample, socioeconomic precarity can further amplify specific discrimination experiences.

### 4.6. Discrimination Burden by Identity Group

**Figure 1:**
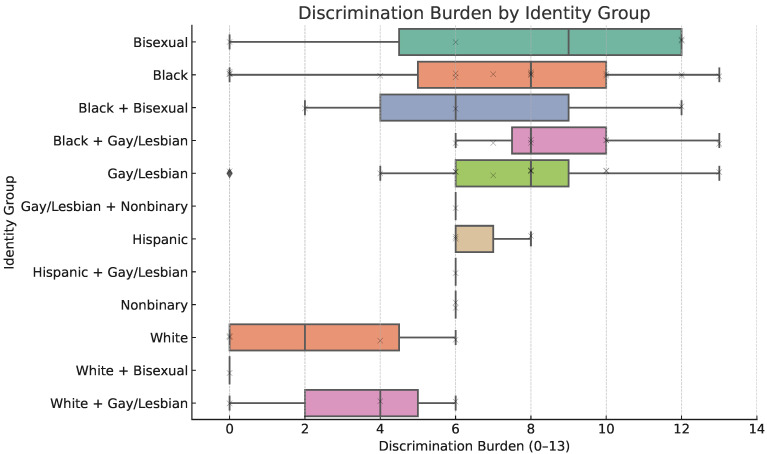
Boxplot showing the distribution of total reported discrimination experiences by intersectional identity group. The burden score reflects the number of distinct discrimination contexts reported (e.g., healthcare, housing, race, sexual orientation). The diamond indicates that at least one participant in that group reported no discrimination burden.

A composite discrimination burden score was calculated for each participant, based on the 33 discrimination types, to capture the cumulative impact of stigma across multiple domains. Figure 1 presents a boxplot of these scores. The median burden was highest among participants identifying as Bisexual, Black, Black and Gay/Lesbian, and Gay/Lesbian, with reported scores extending up to 13 for the latter 3 identity groups. Other intersectional groups such as Black + Bisexual and Gay/Lesbian + Nonbinary also exhibited high median burdens despite smaller sample sizes. The visualized interquartile ranges highlight not only the heightened burden within specific subgroups but also the heterogeneity of stigma among multiply marginalized individuals. These distributions support the concept of cumulative disadvantage linked to intersectional identity and set the stage for a deeper exploration of specific discrimination types and settings.

### 4.7. Discrimination by Type and Setting: Heatmap Synthesis

**Figure 2:**
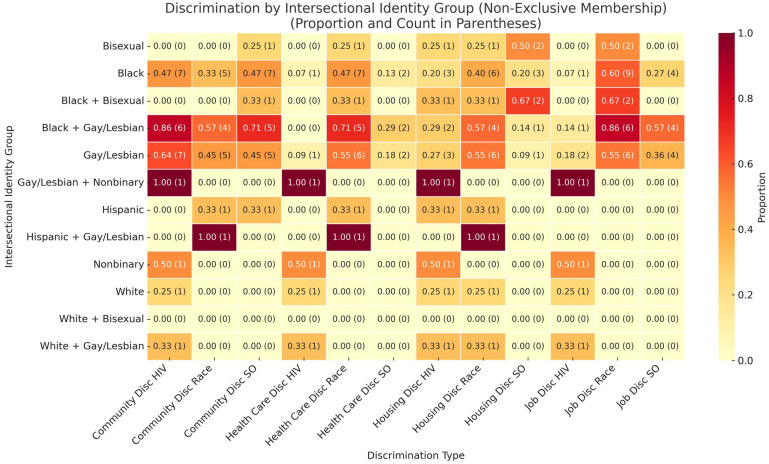
Heatmap of discrimination burden by intersectional identity group. Each cell reflects the proportion and total number of distinct types of discrimination reported by participants within a specific intersectional identity group. Darker shades indicate higher cumulative burden.

The heatmap shown in Figure 2 visually summarizes the total discrimination burden reported by participants, grouped by intersectional identity configurations. Each cell represents the cumulative number of distinct discrimination types (e.g., by HIV status, race, and sexual orientation) experienced by participants within a specific identity group. It is possible for individual participants to be included in more than one group. Darker shades indicate higher total burden. For example, participants identifying as both Black and Gay/Lesbian show consistently darker cells, reflecting a higher reported frequency of discrimination across domains. In contrast, identity groups with fewer intersecting marginalized identities, such as those identifying only as Bisexual, exhibit lighter shading, indicating comparatively lower burden.

The heatmap shows that Black participants report consistently high rates of discrimination across domains, especially in healthcare, community, and job settings. Notably, individuals identifying as both Black and Gay/Lesbian experienced some of the highest proportions of discrimination, particularly related to HIV and race in both community and job discrimination. Hispanic and Bisexual participants also show elevated levels of discrimination, though results are not as meaningful due to very small participation numbers. The heatmap illustrates that discrimination based on sexual orientation (SO) is not limited to one setting but spans community, healthcare, housing, and employment. Overall, the results underscore the cumulative burden of intersectional stigma. Participants with multiple marginalized identities tend to experience discrimination across more domains. This pattern reinforces the QCA findings, illustrating how compounded identities correspond to greater exposure to structural and interpersonal stigma.

### 4.8. Summary

Results from this study highlight a uniformly high burden of discrimination among PLHIV in Southern Illinois. Participants with multiple marginalized identities experienced particularly high levels of stigma, especially in healthcare and community domains. This structural marginalization appears deeply entrenched. The combination of QCA and visual synthesis reveals patterns of intersectional exclusion that can inform targeted, bundled interventions in under-resourced regions.

## 5. Discussion

This pilot study provides preliminary evidence of the pervasive structural and social discrimination faced by PLHIV in Southern Illinois. The uniformly high rates of discrimination reported across all identity groups, combined with significant patterns of compounded burden among multiply marginalized respondents, affirm the value of intersectionality-informed frameworks in HIV research.

The most striking result, that 77% of participants reported experiencing at least one form of discrimination, suggests that systemic stigma is deeply embedded across multiple sectors. This finding resonates with national studies showing that stigma related to HIV, race, sexual orientation, and gender identity intersect to impede care, compromise adherence, and worsen psychosocial outcomes. Our study extends this understanding into a less-studied geographic context where access to affirming care is often limited.

Intersectional analysis revealed that participants with less mainstream or more layered identities tended to report higher burdens. This aligns with prior work emphasizing that structural stigma is not equally distributed, and that gender-diverse individuals often face multiple, concurrent exclusions in healthcare, housing, and community life.

This study also illustrates the utility of QCA as a method for uncovering complex, non-linear relationships in small-N intersectionality research. Unlike variable-centered approaches, QCA enabled us to identify specific configurations of identities, such as Black + Gay/Lesbian or Black + Bisexual, that were associated with consistently high discrimination across multiple domains. This configurational logic reflects real-world complexities, where overlapping forms of marginalization interact rather than act independently. Our findings support the argument that intersectional outcomes cannot be captured through additive models alone and suggest that QCA offers a valuable analytical complement in both qualitative and quantitative HIV stigma research.

Given the methodological challenges of small-N rural samples, this study offers a model for how QCA can be used to extract meaningful intersectional patterns even with limited data. Future studies should consider pairing QCA with qualitative methods to validate and contextualize these patterns, especially in under-researched geographic regions where quantitative approaches may be less feasible.

### 5.1. Illustrative Case Examples

To complement our quantitative findings, we present three anonymized narrative profiles that illustrate the convergence of racial, sexual, gender, and structural vulnerabilities among PLHIV in Southern Illinois. These cases reflect real combinations of characteristics and reported experiences from our dataset and support the major themes identified in our analysis.

#### 5.1.1. Case 1: Shawn—Structural and Social Stigma Across Multiple Fronts

Shawn is a Black Gay man who reports one of the highest discrimination burdens in the dataset. Although his income falls between USD 10,000 and USD 14,999, he experienced housing instability during the COVID-19 pandemic and is currently unemployed. He reports experiencing discrimination in housing due to his HIV status and exclusion in his community based on both his race and HIV status.

In healthcare, Shawn faces racial discrimination, and in social contexts, he experiences stigma related to all three of his marginalized identities: race, sexual orientation, and HIV status. His case reflects how intersectional stigma operates across domains—social, structural, and institutional—and how multiple identity dimensions can compound vulnerability, even when economic status is not the most extreme.

#### 5.1.2. Case 2: Deangelo—Community and Social Isolation Despite Economic Stability

Deangelo, also a Black Gay man, does not report income-related hardship or unemployment, but still experiences substantial stigma. He reports discrimination in healthcare due to race and in the community due to both HIV status and sexual orientation. He also feels socially excluded due to his racial identity.

His case illustrates that intersectional stigma persists independently of income or housing insecurity. Even in the absence of structural poverty, being both Black and Gay while living with HIV produces a complex web of social and community-level marginalization.

#### 5.1.3. Case 3: Tasha—Housing Discrimination at the Intersection of Gender and Sexuality

Tasha identifies as a Black Bisexual woman. Her housing situation worsened during the COVID-19 pandemic, and she reports discrimination in housing related to both her HIV status and her sexual orientation. She also experiences exclusion in her community for the same reasons.

Tasha does not report discrimination in healthcare, but her narrative emphasizes how Bisexuality—especially in women—is often stigmatized in subtle and structural ways. Her case highlights the importance of considering Bisexual identity as a unique vector of stigma that is frequently overlooked in intersectional health research.

### 5.2. Conceptual Synthesis: Intersectional Stigma and Structural Exclusion

**Figure 3:**
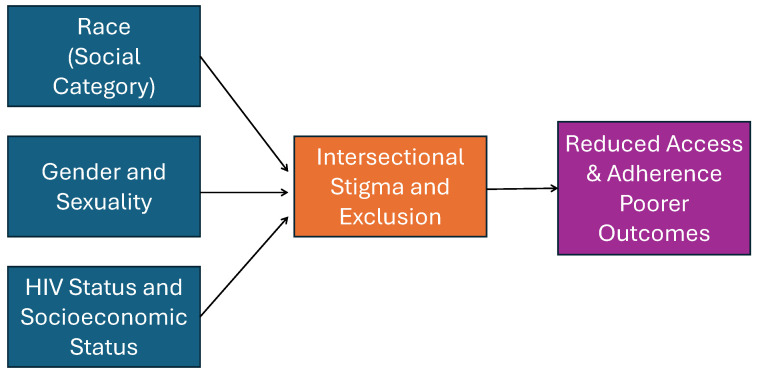
Conceptual model: intersectional identity and structural HIV burden.

Figure 3 offers a synthesized conceptual model derived from our findings. The left side of the model presents three major identity dimensions: race (particularly Black identity), gender/sexual identity (e.g., Nonbinary, Gay/Lesbian, Bisexual), and structural precarity (e.g., low income, HIV status). These dimensions converge to produce “Intersectional Stigma and Exclusion”, a construct supported by our empirical findings and QCA analysis. This central node connects to downstream outcomes, including reduced access to care, lower treatment adherence, and worsened health outcomes. This model offers a synthesis of both theoretical framing and observed data, linking identity structures to systemic health inequities.

### 5.3. Limitations

The primary limitation of this study is its small sample size, which constrains the generalizability of findings and limits the statistical power of subgroup comparisons. Additionally, because of recruitment through HIV service providers and RDS referrals, there is possible self-selection bias. As a cross-sectional survey, the study cannot establish causal relationships. Moreover, all responses were self-reported and subject to recall or social desirability bias. However, the exploratory design and use of pattern detection techniques (e.g., one-hot encoding, QCA-style analysis) were well suited to the goals of early-phase intersectionality research.

### 5.4. Future Directions

Larger, multi-site studies should expand on this pilot to examine regional differences, longitudinal outcomes, and intervention impact. Qualitative and mixed-method approaches can further explore the lived experiences behind the patterns we observe. Importantly, future efforts must remain grounded in the voices and needs of PLHIV themselves, ensuring that research translates into equitable policy and practice.

One particularly striking finding of this study was the “multiplier” effect of being Black, where Black racial identity magnified the effects of other marginalized identities. It remains an open question whether this is unique to being Black, or if other minoritized racial and ethnic groups, such as Latino/Latina populations, would have the same experience. This is a fertile area for future exploration.

In summary, this study highlights that in Southern Illinois, as in many parts of the United States, PLHIV face widespread, multilevel barriers shaped by intersectional stigma and systemic neglect. Tackling these inequities will require more than data—it demands inclusive, participatory approaches and structural reforms that focus on the lived realities of multiply marginalized PLHIV.

## 6. Conclusions

This study contributes to the growing body of literature on intersectionality and structural vulnerability among PLHIV, particularly in rural and under-resourced settings. By applying QCA to a small but diverse sample, we uncovered key identity configurations, such as Black + Bisexual and Black + Gay/Lesbian, that were consistently associated with heightened discrimination across healthcare, housing, and community domains.

Our findings reinforce the argument that stigma is not monolithic but rather shaped by overlapping systems of marginalization. Discrimination was widely reported in this sample, highlighting the saturation of structural and social exclusion in the lives of PLHIV. The prevalence of low income and housing instability further emphasizes the urgency of structural intervention.

Methodologically, this study demonstrates the utility of QCA in exploring complex intersectional dynamics even with small-N datasets. Visual synthesis tools and domain-specific analysis allowed us to convey the nuanced ways in which identity intersects with discrimination.

Policy and programmatic responses should move beyond single-axis interventions. We recommend bundled, intersectionality-informed care models, culturally tailored peer navigation, community-driven stigma reduction, and structural competency training for rural providers. Participatory design must remain central to ensure relevance and sustainability.

Ultimately, dismantling the multilevel barriers that persist for PLHIV will require structural change that is as multifaceted as the stigma it seeks to address. This pilot lays the groundwork for a deeper, more equitable response to the compounded burdens of HIV in rural America.

## Figures and Tables

**Table 1:** QCA-style truth table showing the proportion and number of participants within each identity group reporting different types of discrimination. Rows with zero participants are omitted.

Black	Gay/Lesbian	Bisexual	Nonbinary	Count	Healthcare Race	Healthcare HIV	Housing HIV	Community HIV
0	0	0	0	1	0% (0)	0% (0)	100% (1)	0% (0)
0	0	0	1	1	0% (0)	0% (0)	0% (0)	0% (0)
0	0	1	0	1	0% (0)	0% (0)	0% (0)	0% (0)
0	1	0	0	3	33% (1)	0% (0)	0% (0)	0% (0)
0	1	0	1	1	0% (0)	100% (1)	100% (1)	100% (1)
1	0	0	0	5	20% (1)	20% (1)	0% (0)	20% (1)
1	0	1	0	3	33% (1)	0% (0)	33% (1)	0% (0)
1	1	0	0	7	71% (5)	0% (0)	29% (2)	86% (6)

**Table 2:** QCA-style truth table showing the percentage and count of participants with each combination of identity characteristics who experienced employment or social discrimination. Only combinations with non-zero counts are shown.

Black	Gay/Lesbian	Bisexual	Nonbinary	Count	Employment Discrimination	Social Discrimination
0	0	0	0	1	100% (1)	100% (1)
0	0	0	1	1	100% (1)	100% (1)
0	0	1	0	1	0% (0)	0% (0)
0	1	0	0	3	33% (1)	33% (1)
0	1	0	1	1	100% (1)	100% (1)
1	0	0	0	5	20% (1)	40% (2)
1	0	1	0	3	67% (2)	67% (2)
1	1	0	0	7	100% (7)	86% (6)

**Table 3:** QCA-style truth table: socioeconomic conditions and discrimination outcomes.

Low Income	Housing Unstable	Count	HealthCare Race	HealthCare HIV	Housing HIV	Community HIV
0	0	6	17% (1)	17% (1)	33% (2)	17% (1)
0	1	3	0% (0)	0% (0)	33% (1)	0% (0)
1	0	1	0% (0)	0% (0)	0% (0)	0% (0)
1	1	12	58% (7)	8% (1)	17% (2)	58% (7)

## Data Availability

*Burden of HIV* survey results and analysis materials are freely available, and others are encouraged to use these data. Code used in the original study can be accessed at https://github.com/SIUEComplexNetworksLab/BOHComplexNetworks, accessed on 18 June 2025. Data are available at https://www.openicpsr.org/openicpsr/project/192186/version/V1/view, accessed on 18 June 2025.

## Abbreviations

The following abbreviations are used in this manuscript:

PLHIV	People Living with HIV
QCA	Qualitative Comparative Analysis
SDoH	Social Determinants of Health
PrEP	Pre-Exposure Prophylaxis
MSM	Men who have Sex with Men
RDS	Respondent-Driven Sampling
PWID	People Who Inject Drugs
LGBTQ^+^	Lesbian, Gay, Bisexual, Transgender, and Queer/Questioning,
	Plus other sexual orientations and gender identities
IRB	Institutional Review Board
